# Unlocking the Potential of Na_2_Ti_3_O_7_-C Hollow Microspheres in Sodium-Ion Batteries via Template-Free Synthesis

**DOI:** 10.3390/nano15060423

**Published:** 2025-03-10

**Authors:** Yong-Gang Sun, Yu Hu, Li Dong, Ting-Ting Zhou, Xiang-Yu Qian, Fa-Jia Zhang, Jia-Qi Shen, Zhi-Yang Shan, Li-Ping Yang, Xi-Jie Lin

**Affiliations:** 1 School of Chemistry & Chemical Engineering, Yancheng Institute of Technology, Yancheng 224051, China; sunyg86@iccas.ac.cn (Y.-G.S.); huyu@stu.ycit.edu.cn (Y.H.);; 2 School of Chemistry and Biological Engineering, University of Science and Technology Beijing, Beijing 100083, China; 3 School of Chemistry and Pharmaceutical Sciences, Guangxi Normal University, Guilin 541004, China

**Keywords:** sodium-ion batteries, hollow structure, template-free method, anode materials, Na_2_Ti_3_O_7_-C, cycling stability

## Abstract

Layered sodium trititanate (Na_2_Ti_3_O_7_) is a promising anode material for sodium-ion batteries due to its suitable charge/discharge plateaus, cost-effectiveness, and eco-friendliness. However, its slow Na^+^ diffusion kinetics, poor electron conductivity, and instability during cycling pose significant challenges for practical applications. To address these issues, we developed a template-free method to synthesize Na_2_Ti_3_O_7_-C hollow microspheres. The synthesis began with polymerization-induced colloid aggregation to form a TiO_2_–urea–formaldehyde (TiO_2_-UF) precursor, which was then subjected to heat treatment to induce inward crystallization, creating hollow cavities within the microspheres. The hollow structure, combined with a conductive carbon matrix, significantly enhanced the cycling performance and rate capability of the material. When used as an anode, the Na_2_Ti_3_O_7_-C hollow microspheres exhibited a high reversible capacity of 188 mAh g^−^^1^ at 0.2C and retained 169 mAh g^−^^1^ after 500 cycles. Additionally, the material demonstrated excellent rate performance with capacities of 157, 133, 105, 77, 62, and 45 mAh g^−^^1^ at current densities of 0.5, 1, 2, 5, 10, and 20C, respectively. This innovative approach provides a new strategy for developing high-performance sodium-ion battery anodes and has the potential to significantly advance the field of energy storage.

## 1. Introduction

The growing need for long-lasting electronic systems has driven the search for affordable energy storage technologies. While lithium-ion batteries (LIBs) have been the go-to choice, they are now facing challenges due to the high cost and scarcity of lithium [1,2,3]. Sodium-ion batteries (SIBs) have emerged as a promising alternative, offering similar electrochemical properties to LIBs and benefiting from the abundant supply of sodium [4,5]. They are especially attractive for use in large-scale energy storage [6,7]. The performance of SIBs is critically dependent on the anode materials, which play a key role in determining the battery’s overall capacity. A variety of anode materials, such as metal oxides [8,9], sulfides [10,11], phosphides [12,13], and phosphorus [14,15], have been investigated. However, these materials often experience significant volume changes and lack sufficient pathways for sodium ion transport, resulting in suboptimal rate capabilities and cycling stability [9,16].

Layered sodium trititanate (Na_2_Ti_3_O_7_) is a potential anode material for SIBs, thanks to its low average discharge plateau for reversible Na^+^ storage (approximately 0.3 V versus Na/Na^+^) and its high theoretical Na^+^ storage capacity [17,18]. Despite these advantages, Na_2_Ti_3_O_7_ has issues with poor cycling stability and low electron conductivity, which hinder its practical application. To overcome these challenges, researchers have explored various strategies, including aliovalent doping [19,20], conductive coatings [21,22], and nano-structuring [23,24]. Aliovalent doping with elements like Nb, V, Ti, and F has been shown to improve the cycling stability and electron conductivity of Na_2_Ti_3_O_7_ [25,26,27,28]. For instance, Na_2_Ti_2·97_Nb_0·03_O_7_ [26] demonstrates a higher reversible capacity, better rate performance, and cycling stability compared to undoped Na_2_Ti_3_O_7_. Doping with V [26] leads to a partial reduction of Ti^4^^+^ to Ti^3^^+^, which increases the electron conductivity and enhances the overall electrochemical performance. However, aliovalent doping can introduce lattice defects, negatively affecting the cycling stability of the TiO_6_ layer in Na_2_Ti_3_O_7_.

In recent years, hollow sphere structures have garnered significant attention for their potential to enhance the performance of battery materials [29,30,31,32]. Hollow spheres offer a larger surface area, shorter diffusion paths, and better structural stability, which are beneficial for improving the rate capability and cycle life of anode materials. For example, uniform Na_2_Ti_3_O_7_ hollow spheres assembled from N-doped carbon-coated ultrathin nanosheets have been synthesized, demonstrating the best rate performance ever reported for Na_2_Ti_3_O_7_, with a capacity of over 60 mAh g^−^^1^ after 1000 continuous cycles at a high rate of 50 C [33]. Additionally, a unique red blood cell-like hollow carbon sphere-supported Na_2_Ti_3_O_7_ sodium titanate nanosheet structure (Na_2_Ti_3_O_7_@RHCS) [34] has been designed, which improves electrical conductivity and preserves structural stability, achieving a reversible capacity of 110.46 mAh g^−^^1^ at 5C and 45.71 mAh g^−^^1^ at 50C.

Building on these findings, we developed a novel template-free synthetic methodology for fabricating Na_2_Ti_3_O_7_-C hollow microspheres. This approach avoids the complexities and costs associated with template-based methods, offering a more straightforward and scalable route to producing high-performance anode materials. The synthesis process began with polymerization-induced colloid aggregation to create a TiO_2_–urea–formaldehyde (TiO_2_-UF) precursor, which was then subjected to heat treatment to induce progressive inward crystallization, resulting in the formation of inner voids within the microspheres. The presence of a hollow cavity, coupled with the incorporation of a conductive carbon matrix, significantly enhanced the cycling performance and rate capability of the Na_2_Ti_3_O_7_-C hollow microspheres when used as an anode material. As expected, our results show that the Na_2_Ti_3_O_7_-C hollow microspheres exhibited a reversible capacity of 188 mAh g^−^^1^ at 0.2C in the voltage range of 0.01–2.5 V. Even after 500 cycles, the capacity remained at 169 mAh g^−^^1^. This innovative approach not only provides a new strategy for the development of high-performance SIB anodes but also has the potential to significantly advance the field of energy storage, paving the way for more efficient and sustainable power solutions.

## 2. Materials and Methods

All reagents and chemicals used were of analytical grade and commercially available unless otherwise specified.

### 2.1. Synthesis of TiO_2_-UF Precursor

A solution was prepared by mixing 10 mL of tetrabutyl titanate (TBOT), 60 mL of deionized water, and 1 mL of hydrochloric acid. This mixture was stirred for 30 min at 60 °C in an oil bath. The resultant TiO_2_ sol, with a volume of 10 mL, was then combined with 1 g of urea in 40 mL of deionized water. The pH of the solution was maintained at around 1.5 by the addition of hydrochloric acid. Next, 2 mL of 37 wt% formaldehyde was added while stirring vigorously. The resulting white precipitate was collected via centrifugation, washed sequentially with water and ethanol, and dried overnight at 80 °C for further analysis.

### 2.2. Synthesis of Na_2_Ti_3_O_7_-C Hollow Microspheres

To fabricate the Na_2_Ti_3_O_7_-C hollow microspheres, a blend of the TiO_2_-UF precursor and a sodium source (sodium hydroxide) was prepared with a Na/Ti molar ratio of 2.05:3. This blend is subsequently calcined at 600 °C for 3 h under an argon atmosphere.

For reference, the preparation of Na_2_Ti_3_O_7_ hollow microspheres and Na_2_Ti_3_O_7_-C solid microspheres follow a similar procedure to that of the Na_2_Ti_3_O_7_-C hollow microspheres, with the only difference being the heating of the mixture in an air flow and pH adjustment to 2.

### 2.3. Synthesis of Na_2_Ti_3_O_7_ Hollow Microspheres

The synthesis of the Na_2_Ti_3_O_7_ hollow microspheres follows the same procedure to that of the Na_2_Ti_3_O_7_-C hollow microspheres. The primary difference lies in the calcination atmosphere. Specifically, the blend of TiO_2_-UF precursor and sodium hydroxide (with a Na/Ti molar ratio of 2.05:3) was calcined at 600 °C for 3 h in an air flow instead of an argon atmosphere.

### 2.4. Synthesis of Na_2_Ti_3_O_7_-C Solid Microspheres

The preparation of Na_2_Ti_3_O_7_-C solid microspheres also follows a similar procedure as the hollow microspheres, with key differences in the synthesis of the TiO_2_-UF precursor. Specifically, the TiO_2_-UF precursor for Na_2_Ti_3_O_7_-C solid microspheres is synthesized at pH = 2. This pH condition promotes the formation of dense, solid structures by influencing the polymerization behavior of the precursor.

### 2.5. Characterization

The synthesized Na_2_Ti_3_O_7_-based materials were characterized using a range of techniques to evaluate their phase, structure, morphology, and composition. The crystalline phase and structure were determined using X-ray diffraction (XRD) with a Rigaku Miniflex diffractometer, operating in step-scan mode over the 2*θ* range of 5–70° (scan speed of 2° min^−^^1^) using Cu-Kα radiation (λ = 0.154 nm). The surface morphology and composition of the samples were analyzed using field-emission scanning electron microscopy (FESEM, Hitachi S-4700 FE-SEM, Japan) operating at an acceleration voltage of 10 kV. Transmission electron microscopy (TEM) images and energy-dispersive X-ray (EDX) analysis were obtained and performed using a JEM-2100F (JEOL, Japan) at an accelerating voltage of 200 kV. Thermogravimetric analysis (TGA) was conducted using a SII WXSTAR6000-TGA6300 (Japan) in air at a heating rate of 10 °C min^−^^1^ to analyze the carbon content in the samples. Surface analysis was performed using X-ray photoelectron spectroscopy (XPS, Thermo Scientific ESCALab220i-XL, USA) with an X-ray source of Al Kα. The N_2_ adsorption–desorption isotherm was characterized using an automatic surface analyzer (SSA-7300, China) at a temperature of 77 K. Subsequently, the specific surface area was calculated based on the Brunauer–Emmett–Teller (BET) equations. The Raman spectra were acquired by means of a HORIBA LabRAM HR Evolution spectrometer, which employed an excitation wavelength of 532 nm.

### 2.6. Electrochemical Measurements

The battery performance evaluation of Na_2_Ti_3_O_7_-based materials was conducted using CR2032 coin cells at room temperature. The working electrode was prepared by mixing the active material, carbon black (Super-P), and binder poly(vinylidene fluoride) (PVDF) at a weight ratio of 80:10:10. This mixture was then casted onto a pure copper foil (99.9%) followed by drying at 80 °C for 10 h in a vacuum oven, and cut into circular electrodes with an area of 0.64 cm^2^. The mass loading of the active material was about 1.7 mg cm^−2^. Pure sodium foil was used as the counter and reference electrode, while a glass fiber (Whatman) served as the separator. The electrolyte was composed of 1.0 M NaClO_4_ in a propylene carbonate (PC)/ethylene carbonate (EC) solvent mixture with a volumetric ratio of 1:1. The cell assembly took place in an argon-filled glove box with moisture and oxygen concentrations below 1.0 ppm. Charge/discharge tests and the galvanostatic intermittent titration technique (GITT) were performed using a LAND battery test system with a voltage window set between 0.01 V and 2.5 V. Cyclic voltammogram (CV) curves were obtained using an Autolab electrochemical workstation (PGSTAT 302N) at a scan rate of 0.1 mV s^−1^ within the voltage range of 0.01 V to 2.5 V. Electrochemical impedance spectroscopy (EIS) measurements were conducted over a frequency range of 100 kHz to 100 mHz at room temperature.

## 3. Results and Discussion

**Figure 1:**
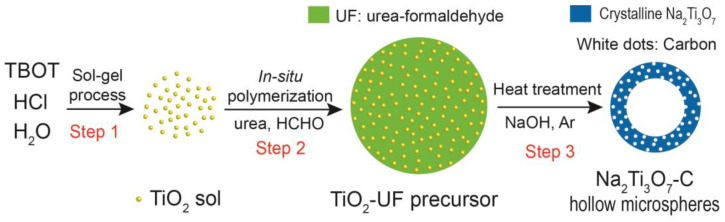
Schematic illustration of the synthesis process for Na_2_Ti_3_O_7_-C hollow microspheres. The process comprises three main steps: (1) the sol–gel process for TiO_2_ sol preparation, (2) in situ polymerization to form the TiO_2_-UF precursor, and (3) controlled heat treatment to induce the formation of void spaces within the particles.

Figure 1 depicts the formation process of Na_2_Ti_3_O_7_-C hollow microspheres. Initially, a TiO_2_ sol was synthesized via a well-established sol–gel technique, involving the hydrolysis and condensation of tetrabutyl titanate (TBOT) in an acidic environment (Step 1). The TEM image in Figure S1 demonstrates that the TiO_2_ sol colloids possess low crystallinity, exhibiting a diameter of approximately 8 nm. Subsequently, a polymerization-induced colloid aggregation process was employed to encapsulate and confine TiO_2_ particles within urea–formaldehyde (UF) resin, yielding TiO_2_-UF microspheres (Step 2). Through a precisely controlled thermal treatment, the TiO_2_ particles undergo crystallization and react with NaOH, resulting in inward crystallization and outward contraction processes, leading to the formation of inner cavities within the microspheres (Step 3). For reference, the preparation of Na_2_Ti_3_O_7_ hollow microspheres and Na_2_Ti_3_O_7_-C solid microspheres followed a similar procedure to that of the Na_2_Ti_3_O_7_-C hollow microspheres, with the only difference being the heating of the mixture in an air flow and pH adjustment to 2.

**Figure 2:**
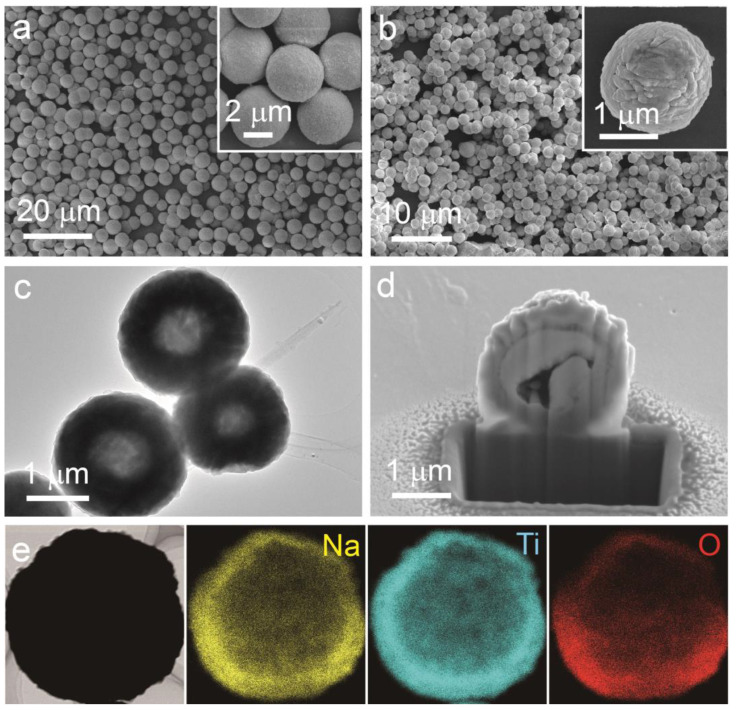
FESEM images of the TiO_2_-UF precursor (**a**) and Na_2_Ti_3_O_7_-C hollow microspheres (**b**). TEM image (**c**), cross-sectional SEM image (**d**), and EDX analysis (**e**) of Na_2_Ti_3_O_7_-C hollow microspheres.

The morphological and structural characteristics of the synthesized materials were thoroughly investigated. The FESEM image (Figure 2a) reveals well-defined spherical TiO_2_-UF precursor particles with a size distribution of approximately 4.5–5 μm, exhibiting rough surface characteristics (inset of Figure 2a). To elucidate the decomposition process of the TiO_2_-UF precursor and quantify the TiO_2_ content, thermogravimetric analysis (TGA) was performed over a temperature range of 20–800 °C (Figure S3). The results indicate a rapid weight loss below 300 °C, corresponding to the decomposition of the UF component, with the weight remaining constant above 600 °C, signifying complete decomposition and a residual TiO_2_ content of 17.3% by mass. Subsequently, the TiO_2_-UF precursor was mixed with NaOH (Na/Ti molar ratio of 2.05:3) under an inert atmosphere to fabricate Na_2_Ti_3_O_7_-C hollow microspheres. The FESEM image (Figure 2b) shows that the resulting Na_2_Ti_3_O_7_-C retains a similar spherical morphology to the precursor but with a reduced diameter. The DLS analysis (Figure S2) confirms that the average diameter of the Na_2_Ti_3_O_7_-C hollow microspheres is approximately 2.5 µm, with a narrow size distribution ranging from 0.8 to 4.0 µm, which is consistent with the SEM image shown in Figure 2b. The TEM image (Figure 2c) demonstrates the presence of void spaces within the core particles of the Na_2_Ti_3_O_7_-C sample, while the cross-sectional SEM image (Figure 2d) further verifies the formation of a hollow cavity. In contrast, the TEM image of Na_2_Ti_3_O_7_-C solid microspheres (Figure S1b) reveals a dense, compact structure without internal voids. Additionally, EDX analysis of randomly selected microspheres (Figure 2e) reveals a uniform distribution of Na, Ti, and O elements across the shell, with the clear delineation of the hollow structure confirming its successful formation. This hollow architecture is attributed to the controlled heat treatment of the inorganic–organic precursor, which forms microspheres through a polymerization-induced colloid aggregation process, as demonstrated in our previous work [35,36].

**Figure 3:**
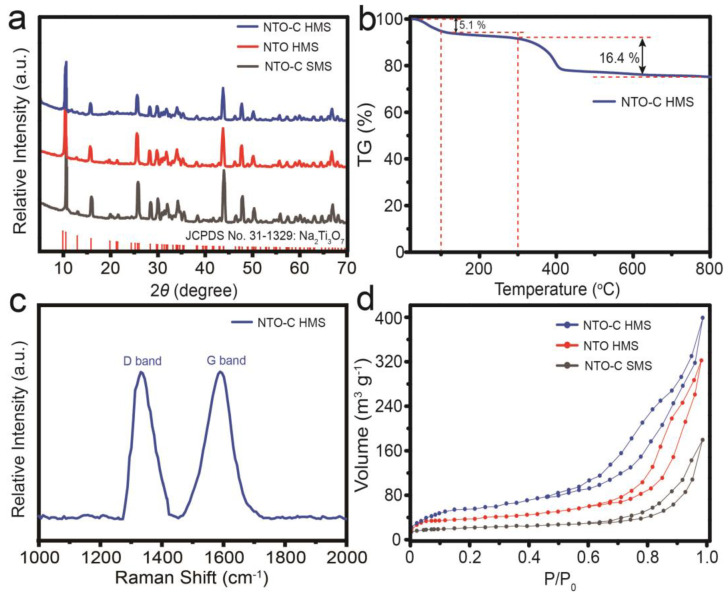
(**a**) XRD patterns of NTO-C SMSs (gray line), NTO HMSs (red line), and NTO-C HMSs (blue line). (**b**) TGA curve of NTO-C HMSs over the range of 20–800 °C in air. (**c**) Raman spectrum of NTO-C HMSs. (**d**) N_2_ adsorption–desorption isotherms of the as-prepared samples.

The XRD patterns of the Na_2_Ti_3_O_7_-C hollow microspheres (denoted as NTO-C HMSs), Na_2_Ti_3_O_7_ hollow microspheres (denoted as NTO HMSs), and Na_2_Ti_3_O_7_-C solid microspheres (denoted as NTO-C SMSs) are shown in Figure 3a. Despite the low crystallinity of TiO_2_ in the TiO_2_-UF precursor (Figure S4), the diffraction peaks after heat treatment align with the standard card of Na_2_Ti_3_O_7_ (JCPDS No. 31-1329). The absence of impurity peaks corresponding to the anatase or rutile phases of TiO_2_ confirms the high purity of the Na_2_Ti_3_O_7_ crystal structure. However, Na_2_Ti_3_O_7_, characterized by a large bandgap of 3.7 eV [36], is inherently an insulator, which significantly hampers electron transfer and results in poor rate performance. To address these limitations, carbon modification was employed to enhance the conductivity and overall electrochemical performance of Na_2_Ti_3_O_7_. Carbon modification is a widely used strategy to improve the electrochemical properties of materials by providing a conductive matrix that facilitates electron transfer [22,23]. To analyze the carbon content in Na_2_Ti_3_O_7_-C hollow microspheres, thermogravimetric analysis (TGA) was conducted as shown in Figure 3b, revealing a carbon content of approximately 16.4%. This carbon content is crucial for enhancing the conductivity of the material. Furthermore, Raman spectroscopy (Figure 3c) was employed to assess the degree of graphitization of the carbon in NTO-C HMSs. The intensity of the G band, indicative of graphitic carbon atoms, is comparable to the intensity of the D band associated with disordered carbon atoms. This indicates a well-graphitized carbon matrix, which is essential for improving the electrochemical performance of the material. It is commonly believed that higher temperatures promote the graphitization of carbon, thereby enhancing its conductivity. However, excessively high temperatures can induce structural changes in Na_2_Ti_3_O_7_, which may adversely affect its electrochemical performance. Based on these, we considered the balance between carbon graphitization and the structural integrity of Na_2_Ti_3_O_7_. Therefore, we chose the optimal temperature of 600 °C for synthesis that maximizes the benefits of carbon modification without compromising the material’s structural stability. Our synthesis method ensures that the carbon content is distributed within the material, forming a conductive matrix that facilitates electron transfer and overall electrochemical performance. Additionally, Brunauer–Emmett–Teller (BET) surface area analysis was performed to evaluate the specific surface area of the materials. As depicted in Figure 3d, the BET surface area of NTO-C HMSs was found to be 223 m^2^ g^−^^1^, significantly higher than that of NTO-C SMSs (65 m^2^ g^−^^1^) and NTO HMSs (119 m^2^ g^−^^1^). The higher specific surface area of NTO-C HMSs is attributed to their hollow structure and porous carbon matrix, which facilitate electrolyte penetration and the transfer of electrons and ions.

**Figure 4:**
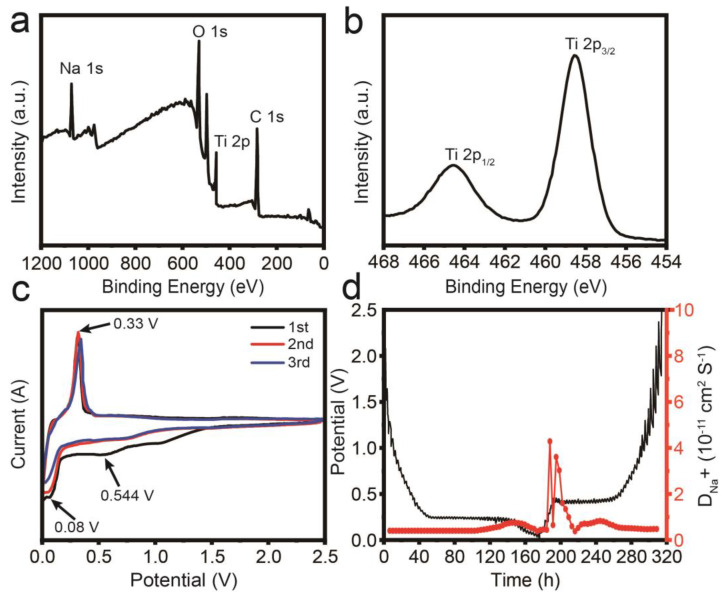
(**a**) Full XPS spectrum and (**b**) high-resolution Ti 2p spectra of NTO-C HMSs. (**c**) CV curves of NTO-C HMSs at 0.1 mV s^−^^1^ within 0.01 V to 2.5 V. (**d**) GITT curves for NTO-C HMSs during the first cycle.

The chemical valence states of NTO-C HMSs were investigated through X-ray photoelectron spectroscopy (XPS). The full XPS survey spectrum (Figure 4a) confirms the presence of Na, Ti, O, and C elements. The high-resolution Ti 2p spectrum (Figure 4b) shows two peaks at 464.5 eV and 458.6 eV, corresponding to Ti 2p_1/2_ and Ti 2p_3/2_, respectively, confirming the presence of Ti^4^^+^ in NTO-C HMSs. The sodium storage potential of hollow microspheres composed of Na_2_Ti_3_O_7_-C is examined as an anode material for sodium-ion batteries in this study. Figure 4c illustrates the cyclic voltammetry (CV) curves for the Na_2_Ti_3_O_7_-C hollow microspheres over multiple cycles within the voltage range of 0.01–2.5 V (vs. Na/Na^+^) at a scan rate of 0.1 mV s^−1^. The first cathodic process reveals a peak at 0.544 V, which corresponds to the irreversible formation of a solid electrolyte interphase (SEI) layer. This peak diminishes in subsequent cycles, with the cyclic curves overlapping, indicating the irreversibility of the SEI layer. Another consistent peak at 0.08 V emerges during all cathodic cycles, signifying the insertion of Na^+^, while the oxidation peak at 0.33 V corresponds to the extraction of Na^+^ from the NTO-C HMS electrode. From the second cycle onward, the curves overlap almost completely, indicating excellent electrochemical reversibility of the NTO-C HMS electrode. The diffusion properties of Na^+^ in NTO-C HMSs were studied using the galvanostatic intermittent titration technique (GITT), as shown in Figure 4d. By analyzing the GITT curves under different states, the average Na^+^ diffusion coefficients were calculated to be 6.5 × 10^−^^12^ cm^2^ s^−^^1^ during discharge and 7.3 × 10^−^^12^ cm^2^ s^−^^1^ during charge. The hollow structure of NTO-C HMSs provides a more favorable pathway for sodium ion transport. Moreover, the presence of the hollow cavity significantly shortens the diffusion path of Na^+^, thereby effectively enhancing the charge transport kinetics within the material.

**Figure 5:**
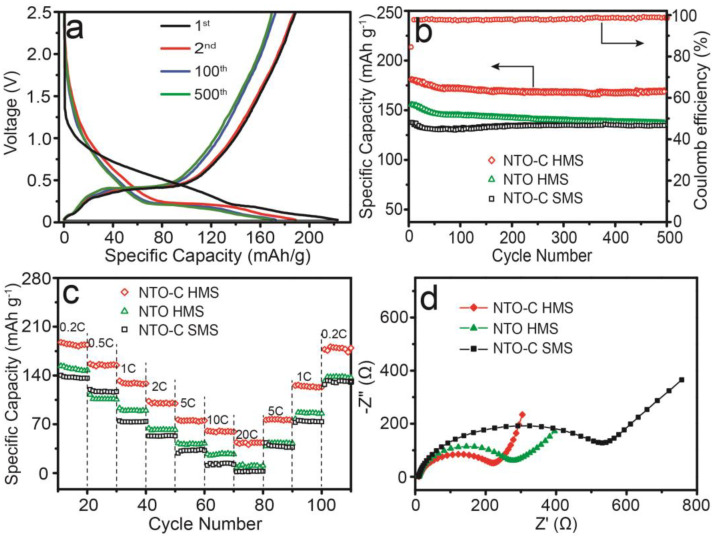
(**a**) Charge/discharge profiles of Na_2_Ti_3_O_7_-C hollow microspheres at different cycles with a current density of 0.2C. (**b**) Comparison of cycling stability for the prepared samples at 0.2C (1C = 177 mA g^−^^1^). Rate capability (**c**) and EIS spectra (**d**) of NTO-C SMSs, NTO HMSs, and NTO-C HMSs.

Figure 5a presents the representative charge/discharge profiles of NTO-C HMSs for different cycles (1st, 2nd, 100th, and 500th) at a current density of 0.2C. The discharge specific capacity in the first cycle reaches 223.1 mAh g^−^^1^, exceeding the theoretical capacity due to contributions from the carbon content and the decomposition of the electrolyte, as well as the formation of the SEI layer. The charge specific capacity is 188.4 mAh g^−^^1^, resulting in an initial Coulombic efficiency (CE) of 84.4%, attributed to the irreversible reactions associated with SEI layer formation. Subsequently, the charge/discharge curves exhibit plateau regions around 0.45 V and 0.24 V and remain nearly identical after 500 cycles, consistent with the CV results. To facilitate comparison, the electrochemical performance of NTO HMSs and NTO-C SMSs was also evaluated. Interestingly, NTO-C HMSs demonstrated negligible capacity degradation over extended cycling, with a reversible capacity of 169.2 mAh g^−^^1^ after 500 cycles (Figure 5b), corresponding to a capacity retention rate of 89.8%. In contrast, the initial capacities of the NTO HMSs and NTO-C SMSs at 0.2C were 155 mAh g^−^^1^ and 141 mAh g^−^^1^, respectively. The enhanced capacity and cyclability of NTO-C HMSs can be attributed to their hollow structure and carbon matrix, which increases the contact area with the electrolyte and reduces diffusion paths of electrons and ions. Furthermore, the carbon matrix not only provides a conductive network that facilitates electron transfer but also improves the structural stability of the material. The uniform distribution of elements within the hollow microspheres, as evidenced by EDX mapping (Figure 2e), ensures efficient sodium ion transport and interaction with the carbon matrix, contributing to the overall electrochemical performance. The hollow structure provides space for volume expansion during sodium intercalation/deintercalation, thereby reducing mechanical stress on the electrode particles.

Moreover, the rate capability tests further underscore the benefits conferred by the hollow structure and conductive carbon matrix. As shown in Figure 5c, NTO-C HMSs exhibited enhanced rate performance with reversible specific capacities of 188, 157, 133, 105, 77, 62, and 45 mAh g^−^^1^ at current densities of 0.2, 0.5, 1, 2, 5, 10, and 20C, respectively. Notably, the capacity fully recovered to its initial value when the current density was reduced back to 0.2C, highlighting the excellent reversibility of the electrode material. In contrast, NTO HMSs and NTO-C SMSs showed inferior rate performance, underscoring the importance of the hollow structure and carbon matrix in enhancing the electrochemical properties of Na_2_Ti_3_O_7_ for SIBs. As sodium ions intercalate and deintercalate, the hollow architecture provides space for volume expansion, thereby reducing mechanical stress on the electrode particles. This structural benefit is further enhanced by the carbon matrix, which not only improves conductivity but also provides mechanical stability. The combination of these factors results in superior cycling stability and rate performance, as demonstrated by the negligible capacity degradation over 500 cycles and the excellent reversibility observed in rate capability tests (Figure 5c). To gain a deeper understanding of the kinetics during the Na^+^ charge/discharge process, electrochemical impedance spectroscopy (EIS) analysis was conducted. The Nyquist plots (Figure 5d) reveal a charge transfer resistance (R_ct_) of 216 Ω for NTO-C HMSs. This lower R_ct_ indicates that the charge transfer interface reaction is easier for NTO-C HMSs compared to other variants, thereby elucidating the enhanced sodium storage performance. The frequency-dependent behavior, as observed in the impedance spectra, suggests that the carbon matrix and hollow structure facilitate faster ion transport kinetics. This is particularly evident at higher frequencies, where the material exhibits lower impedance, indicating efficient electron and ion transfer.

## 4. Conclusions

In summary, this study successfully synthesizes Na_2_Ti_3_O_7_-C hollow microspheres using a template-free approach, demonstrating their potential as high-performance anode materials for sodium-ion batteries. The hollow structure and conductive carbon matrix significantly enhance the material’s electrochemical properties, including a high specific surface area (223 m^2^ g^−^^1^), excellent rate capability (with reversible capacities ranging from 188 mAh g^−^^1^ at 0.2C to 45 mAh g^−^^1^ at 20C), and superior cycling stability (169.2 mAh g^−^^1^ after 500 cycles at 0.2C with an 89.8% capacity retention rate). These features collectively address the inherent limitations of Na_2_Ti_3_O_7_, such as poor conductivity and structural instability, thereby improving its practical applicability in sodium-ion batteries. The findings highlight the importance of structural engineering in optimizing electrode performance. The template-free synthesis method offers a scalable and cost-effective alternative to conventional templating techniques, while the incorporation of a conductive carbon matrix and hollow architecture provides an efficient framework for sodium ion transport and charge storage. This work not only advances the understanding of Na_2_Ti_3_O_7_-based materials but also provides a general strategy for developing high-performance anodes for next-generation energy storage systems.

## Data Availability

Data are contained within the article.

## Supplementary Materials

The following supporting information can be downloaded at: https://www.mdpi.com/article/10.3390/nano15060423/s1, Figure S1. (a) TEM image and HRTEM image of TiO_2_ sol. The TEM image shows that TiO_2_ sol is irregular colloids and HRTEM image shows it is low crystallinity. (b) TEM image of Na_2_Ti_3_O_7_-C solid microspheres. Figure S2. Dynamic Light Scattering (DLS) Analysis of Na_2_Ti_3_O_7_-C hollow microspheres. Figure S3. TGA curve of TiO_2_-UF microspheres over range of 20–800 °C in air with a heating rate of 10 °C/min. The TGA curve shows that there is a sharply weight loss. Figure S4. XRD patterns of TiO_2_-UF precursor.
