# Supplementary material for: Unlocking the Potential of Na2Ti3O7-C Hollow Microspheres in Sodium-Ion Batteries via Template-Free Synthesis
Source: Nanomaterials (Basel). 2025 Mar 10;15(6):423. doi: 10.3390/nano15060423 (PMC11946267; doi:10.3390/nano15060423)
Supplement: Supplementary file 1 [file nanomaterials-15-00423-s001.zip › nanomaterials-3505193-supplementary.pdf]

Supporting information

Unlocking the Potential of $Na_2Ti_3O_7$-C Hollow Microspheres in Sodium-Ion Batteries via Template-Free Synthesis

Yong-Gang Sun [1], Yu Hu [1], Li Dong [1], Ting-Ting Zhou [1], Xiang-Yu Qian [1], Fa-Jia Zhang [1], Jia-Qi Shen [1], Zhi-Yang Shan [1], Li-Ping Yang [1,2,*] and Xi-Jie Lin [3,*]

[a] *School of Chemistry & Chemical Engineering, Yancheng Institute of Technology, Yancheng 224051, China*

[b] *University of Science and Technology Beijing, Beijing 100083, China*

[c] *School of Chemistry and Pharmaceutical Sciences, Guangxi Normal University, Guilin 541004, China*

*Corresponding Author: yanglp0803@163.com; linxijie@mailbox.gxnu.edu.cn

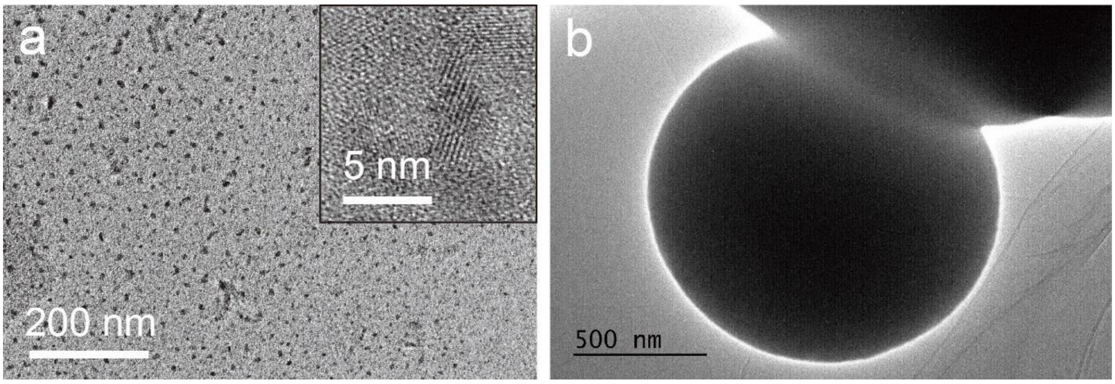

Figure S1 (a) TEM image and HRTEM image of TiO_2 sol. The TEM image shows that TiO_2 sol is irregular colloids and HRTEM image shows it is low crystallinity. (b) TEM image of $Na_2Ti_3O_7$-C solid microspheres.

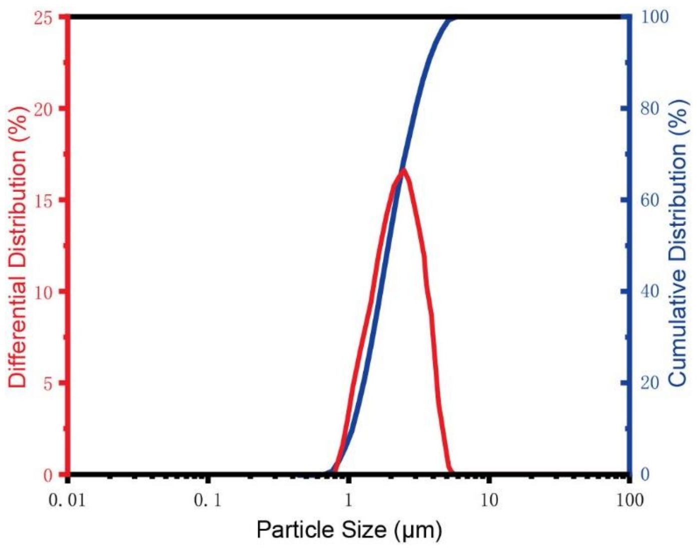

Figure S2 Dynamic Light Scattering (DLS) Analysis of $Na_2Ti_3O_7$-C hollow microspheres.

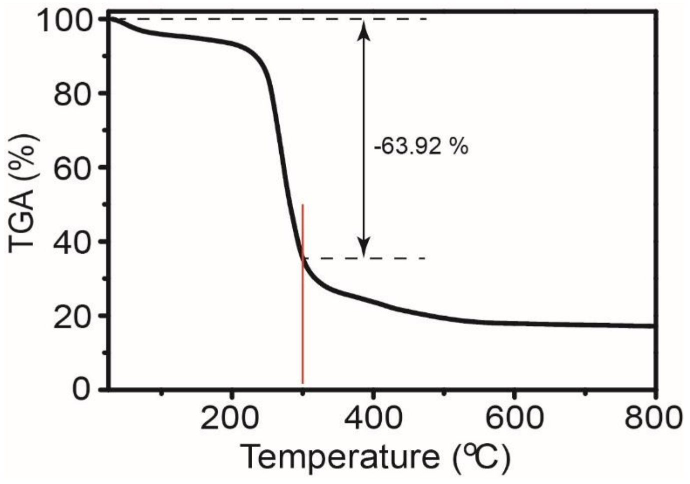

Figure S3 TGA curve of TiO_2-UF microspheres over range of 20-800 ^oC in air with a heating rate of 10 ^oC/min. The TGA curve shows that there is a sharply weight loss.

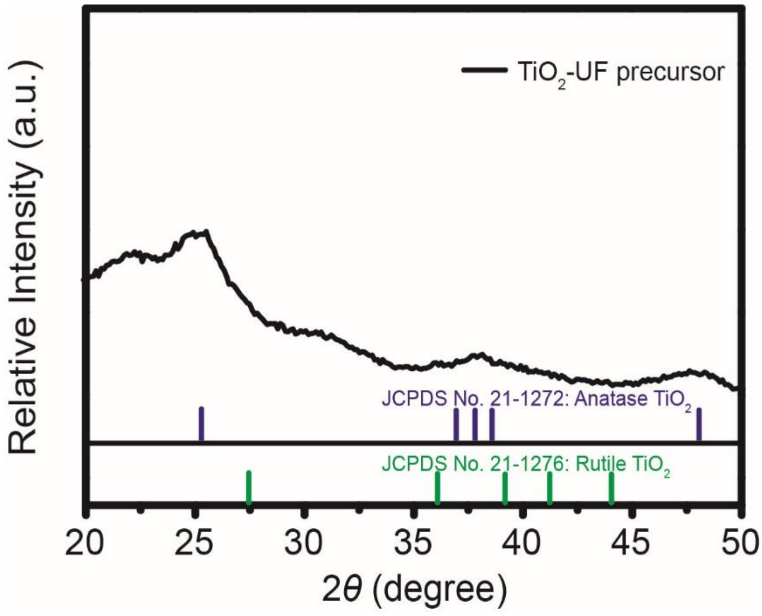

Figure S4 XRD patterns of TiO_2-UF precursor
